# Assessment of Patient‐Reported Outcome Measures in Patients Undergoing Bowel Preparation With Mannitol for Colonoscopy: The SATISFACTION Study

**DOI:** 10.1002/jgh3.70237

**Published:** 2025-08-07

**Authors:** Gian Eugenio Tontini, Cristiano Spada, Peter Uebel, Renato Cannizzaro, Giorgio Ciprandi, Maurizio Vecchi

**Affiliations:** ^1^ Department of Pathophysiology and Organ Transplantation University of Milan Milan Italy; ^2^ Gastroenterology and Endoscopy Unit Fondazione IRCCS Ca' Granda Ospedale Maggiore Policlinico Milan Italy; ^3^ Digestive Endoscopy Unit Fondazione Policlinico Universitario Agostino Gemelli IRCCS Rome Italy; ^4^ Fondazione Poliambulanza Istituto Ospedaliero Brescia Italy; ^5^ Praxis für Gastroenterologie Und Fachärztliche Innere Medizin Im Haus der Gesundheit Ludwigshafen Germany; ^6^ Oncological Gastroenterology Centro di Riferimento Oncologico di Aviano (CRO), National Cancer Institute, IRCCS Aviano Italy; ^7^ Outpatient Department Casa di Cura Villa Montallegro Genoa Italy

**Keywords:** bowel preparation, colonoscopy, oral mannitol, patient‐reported outcomes

## Abstract

**Background:**

Bowel preparation for colonoscopy causes significant discomfort to patients. Large volumes, unpleasant taste, and split‐dosing are the main disadvantages of most laxatives. Oral mannitol could be an attractive option because it acts quickly, requires low volumes, and has a pleasant taste.

**Aims:**

A Phase III study (SATISFACTION) compared oral mannitol with polyethylene glycol‐ascorbate (PEG‐ADC). This *post hoc* analysis investigated the effects on patient‐reported outcomes (PROMs), mainly concerning the perception of using the preparations.

**Methods:**

The SATISFACTION Phase III study was an international, multicenter, randomized (1:1), parallel‐group, endoscopist‐blinded, non‐inferiority trial. Taste of preparations, ease of use, and willingness to repeat the preparation with the same product were scored and assessed by the patients.

**Results:**

Bowel preparation with oral mannitol resulted in a better patient satisfaction profile for all PROMs evaluated.

**Conclusion:**

Oral mannitol for bowel preparation achieved high patient satisfaction and might be a valuable option for colonoscopy.

**Trial Registration:**

ClinicalTrials.gov (https://clinicaltrials.gov/ct2/show/NCT04759885)

## 1 Introduction

Colonoscopy is currently the gold‐standard procedure for investigating and evaluating colonic diseases [1]. However, optimal colon visualization depends on complete bowel evacuation before the process [2]. Adequate bowel preparation is defined by the ability to identify small adenomas (< 5 mm) and/or achieve sufficient validated scores [3]. Conversely, inadequate bowel preparation implies an incomplete procedure and the need to repeat the colonoscopy. Inadequate preparation may also lead to missed cancer or pre‐cancer diagnoses and increase complications and costs [4, 5]. Notably, up to 25% of patients present with inadequate bowel preparation [6].

Preparation for a colonoscopy is a disagreeable and poorly tolerated procedure and constitutes the primary barrier to performing this examination. Many patients report that bowel preparation is the most unpleasant part of the entire investigation, and consequently, many people tend to avoid colonoscopies [7, 8].

Inadequate bowel preparation may depend on several factors, including the taste of laxatives, the large volume of ingested medication solutions, the demanding schedule, physical discomfort, and inadequate explanation (many patients receive nothing more than a reminder).

Accordingly, the tolerability of bowel preparation is of fundamental importance for colonoscopy, and a reasonable level of patient satisfaction improves willingness to undergo the procedure again, thereby maximizing adherence to screening and follow‐up programs [8].

The patient's point of view thus plays a crucial role in colonoscopy management and should guide the choice of the best preparation for the sake of good clinical practice. In addition, patient‐centeredness should be the primary goal of personalized medicine and a key measurement in clinical trials that investigate bowel preparations [9]. In this regard, adherence to the procedure, taste of medications, ease of use, willingness to repeat the preparation, and satisfaction with the preparation are relevant endpoints. The SATISFACTION study investigated the efficacy and safety of oral mannitol for bowel preparation, addressing these issues [10, 11, 12, 13]. This *post hoc* analysis evaluated a series of patient‐reported outcome measures (PROMs) related to acceptability for mannitol and the standard preparation, polyethylene glycol‐ascorbate (PEG‐ASC). In particular, this new analysis aimed to provide a novel elaboration and interpretation of the findings collected in Phase III of the trial, utilizing an original biostatistical approach. As a result, this new analysis would offer outcomes highlighting the relevance of the patient's perspective during bowel preparation, thereby emphasizing the importance of preparation characteristics in the context of Patient‐centered Medicine [14].

## 2 Materials and Methods

### 2.1 Study Design

**FIGURE 1:**
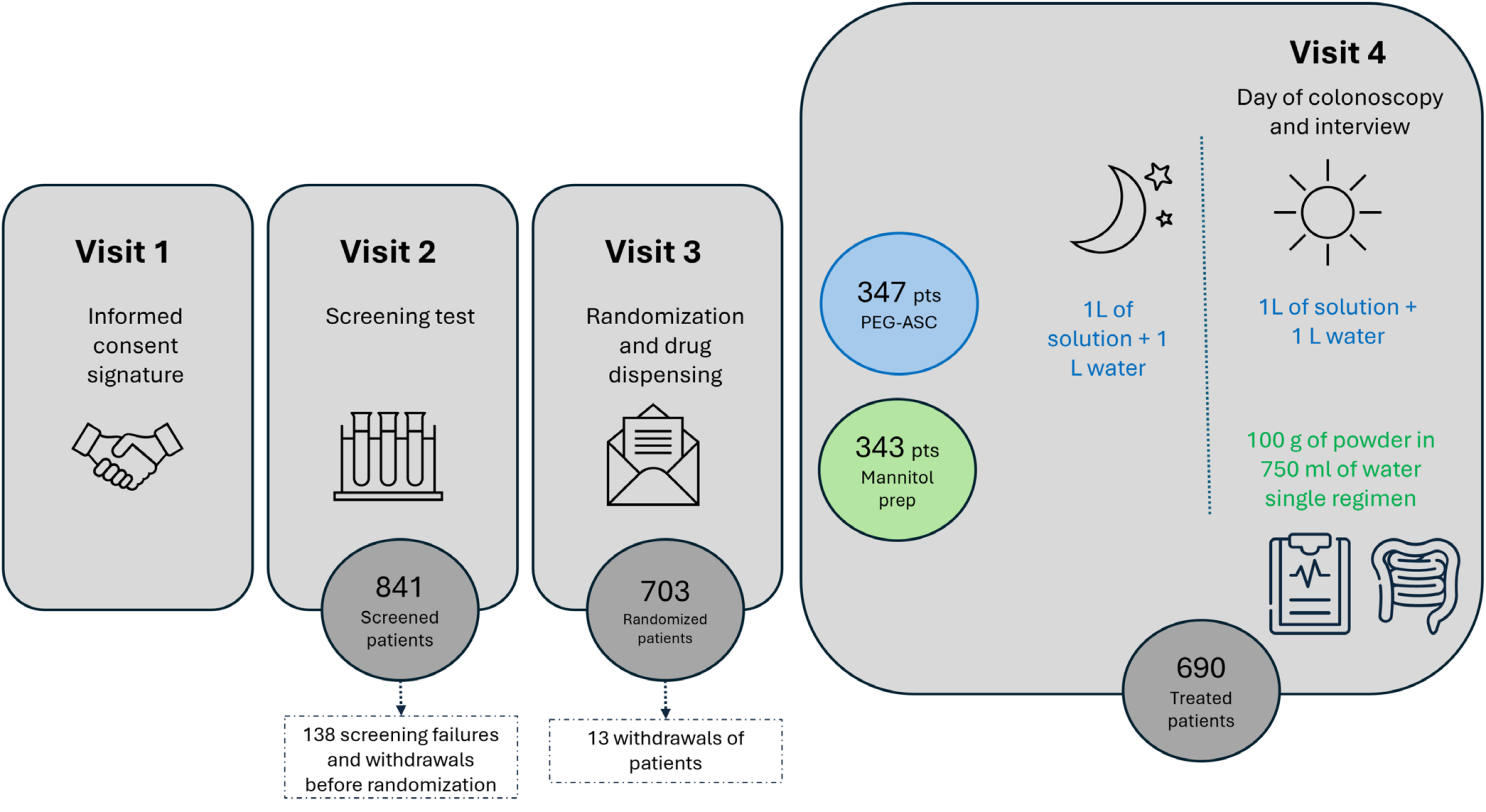
Study design.

The SATISFACTION study was an international, multicenter, randomized, parallel‐group, endoscopist‐blinded trial. It was conducted in 30 centers located in Italy, Germany, France, and Russia. The trial protocol was registered with ClinicalTrials.gov (https://clinicaltrials.gov/ct2/show/NCT04759885) and with EudraCT (eudract_number:2019–002856‐18). The design of the study is summarized in Figure 1.

The patients enrolled were randomized in a 1:1 ratio to one of the following treatment groups:

– mannitol powder 100 g dissolved in 750 mL water to be drunk in 30 min on the day of the colonoscopy, with administration completed at least 4 h before the colonoscopy.
– 2 L PEG‐ASC (Moviprep) administered according to a split‐dose regimen: the first liter over 1–2 h the evening before colonoscopy, and the second liter the morning of the procedure, with administration completed at least 4 h before the colonoscopy.

The complete study methods were reported in detail in recent publications [3, 10, 11, 12, 13].

This post hoc analysis evaluated a series of acceptability outcomes using a new biostatistical approach.

### 2.2 Adherence and Acceptability Endpoints

– Taste: numeric rating scale (NRS) (0 = terrible to 10 = very good).
– Ease of use: NRS (0 = very difficult to 10 = very easy).
– Willingness to repeat the preparation with the same product, stratifying patients into two subgroups, with or without previous colonoscopy.

### 2.3 Statistical Analysis

The analysis of considered variables was performed on the SAFETY SET, which includes all randomized patients who take the study preparation, even if only partially.

The taste and ease of use were analyzed considering the cumulative density of patients for each single score (0–10). Data were expressed using a comparative slope.

The analysis of ‘Willingness to repeat the preparation with the same product’ was conducted in the SAFETY SET by evaluating first the patients with prior colonoscopies, and subsequently those without prior colonoscopies.

Descriptive statistics were applied on willingness to re‐use (yes or no). All analyses were performed using SAS version 9.4.

## 3 Results

The study included 690 patients belonging to the SAFTY SET. Two of them, both in the mannitol group, did not reply to the PROMs interview, so the final count is 688. The demographic data are reported in Table 1.

**TABLE 1:** Demographic characteristics of enrolled patients (safety set).

	Mannitol (*N* = 343)	PEG‐ASC (*N* = 347)	Total (*N* = 690)
Age (years)			
Mean (SD)	54.7 (12.73)	54.6 (12.76)	54.7 (12.74)
Min; Max	20; 84	19; 86	19; 86
Sex, *n* (%)			
Male	130 (37.90)	153 (44.09)	283 (41.01)
Female	213 (62.10)	194 (55.91)	407 (58.99)
Ethnicity, *n* (%)			
Hispanic or latino	3 (0.87)	4 (1.15)	7 (1.01)
Not hispanic or latino	335 (97.67)	340 (97.98)	675 (97.83)
Unknown	5 (1.46)	3 (0.86)	8 (1.16)

*Note:* Percentages were computed on patients in the randomized set.

### 3.1 Ease of Use and Taste

For the easy to use and taste NRS, the data are considered in terms of cumulative density curves.

**FIGURE 2:**
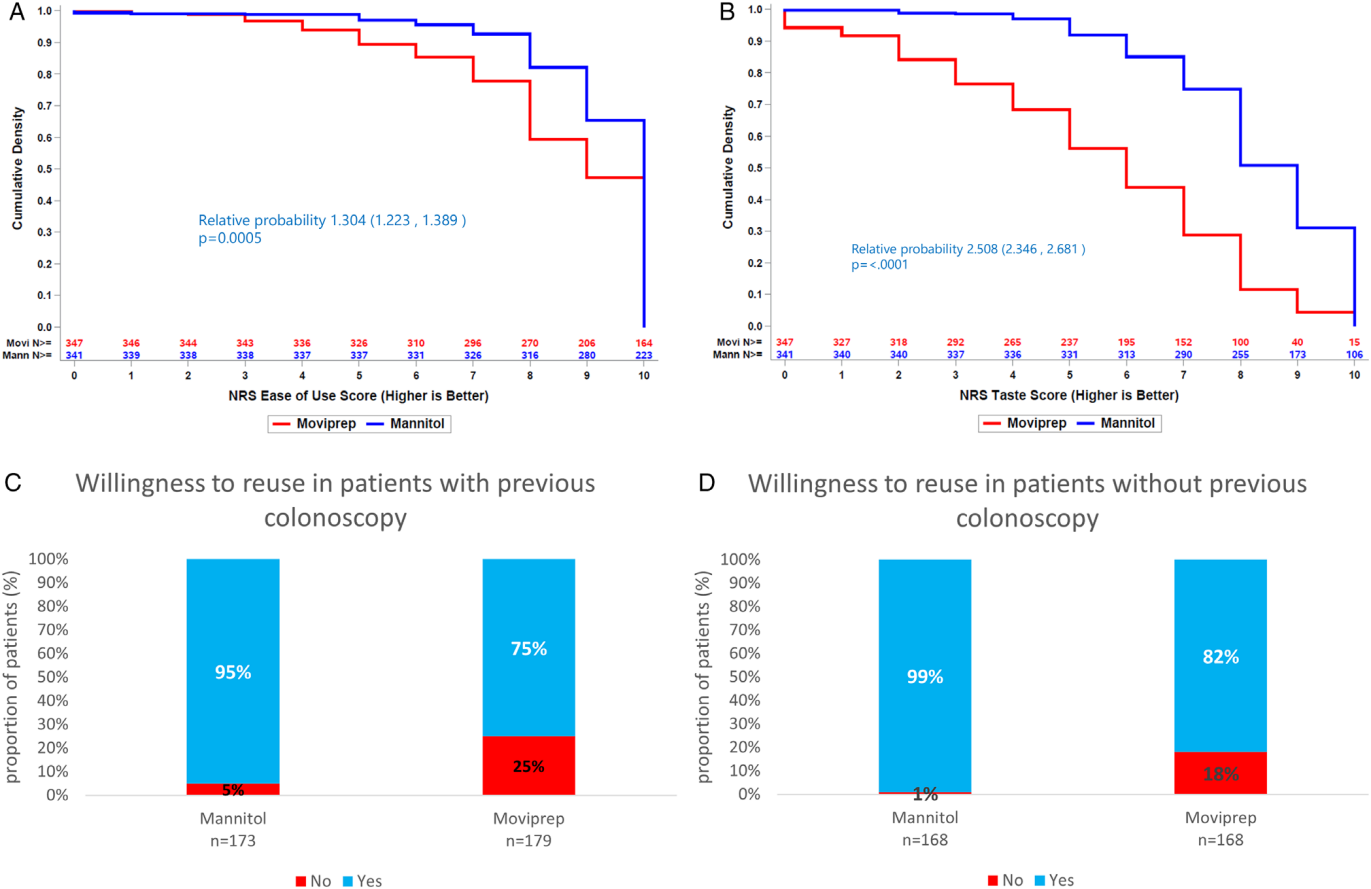
Acceptability endpoints. Panel A: Ease of use; panel B: Taste; panel C: Willingness to reuse the study preparation in patients with previous colonoscopy; panel D: Willingness to reuse the study preparation in patients without previous colonoscopy. “ease of use” and “taste” were assessed within the SAFETY SET. Notably, two patients in the Mannitol group did not complete the questionnaire; therefore, the graph reflects responses from 688 patients. Willingness to reuse percentages was computed on patients with and without previous colonoscopy belonging to the Safety set who have been administered a questionnaire on adherence and acceptability.

For ease of use scores (Figure 2 panel A), mannitol always has a higher fraction of patients achieving that score, or higher compared to Moviprep. The relative probability that mannitol results in a higher ease of use score than Moviprep is approximately 1.30 fold (CI 1.22–1.39, *p* < 0.001); hence a patient is approximately 1.3 fold more likely to achieve a better ease of use score with mannitol compared to Moviprep.

Also for any taste score (Figure 2 panel B) mannitol has a higher fraction of patients achieving that score, or higher, compared to Moviprep. The relative probability that mannitol results in a higher taste score than Moviprep is approximately2.5 fold (VI 2.3–2.70, *p* < 0.0001); therefore, a subject is approximately2.5 fold more likely to achieve a better taste score with mannitol compared to Moviprep.

### 3.2 Willingness to Re‐Use the Study Preparation

For willingness to re‐use, patients were subdivided into two subgroups in relation to having undergone previous colonoscopies. In patients with a previous colonoscopy (Figure 2 panel C), a significantly higher percentage of patients treated with mannitol (95%) would re‐use it than patients treated with PEG‐ASC (95% vs. 75%, *p* < 0.0001).

Also in patients without a previous colonoscopy, there was a significant difference in willingness to re‐use the same drug between the two treatment groups (99% for mannitol vs. 82% for PEG_ASC, *p* < 0.0001): as represented in Figure 2 panel D.

## 4 Discussion

The SATISFACTION study was designed to establish the degree of efficacy and safety of mannitol by comparing it with a reference drug (PEG‐ASC). The study design also considered many aspects of patient satisfaction, as reflected in the trial's title. However, today, medicine is increasingly focused on the patient, as their point of view and engagement are crucial for sharing treatment and adhering to prescribed therapies [15]. Even the WHO recommends the importance of placing the patient at the center of medical care and personalizing treatment. From this point of view, PROMs currently play a significant role in medical care [16, 17], measuring various aspects, including health‐related quality of life, symptoms, functional status, and overall well‐being. PROM data may be used to monitor the progress of individual patients [18], investigate the effects of medical and surgical interventions [19], and improve communication between patients and providers [20]. In this context, preparation for a colonoscopy is a prime example of the importance of the PROMs related to patient acceptability. The most unpleasant aspect of the examination is the preparation itself, considering the taste, the difficulty in preparing the solutions, the high volume of liquids to drink, and the need to empty the bowels frequently, accompanied by colic pain. For this reason, it is essential that the patient can take a preparation that is pleasant‐tasting, easy to administer, has a rapid onset of action (thus allowing a single administration on the morning of the examination), and does not cause particular intestinal discomfort. It is for this reason that the study comparing the handpiece and PEG‐ASC was designed.

This post hoc analysis on PROMS related to colonoscopy preparation acceptability confirmed that mannitol is preferable to PEG‐ASC because the patients who took mannitol were more satisfied than those who took PEG‐ASC. Taste, ease of use, and willingness to re‐use the same product are crucial characteristics for achieving optimal adherence to the preparation, thereby ensuring a valuable colonoscopy.

The SATISFACTION study had some limitations, mainly the lack of a double‐blind design. This aspect was impossible due to different volume and scheduling requirements, such as single‐dose vs. split‐dose. On the other hand, randomization, the inclusion of a control group, and sample size ensured a robust methodology.

In conclusion, oral mannitol for bowel preparation resulted in improved patient satisfaction and may be a new valuable option for colonoscopy preparation.

## Ethics Statement

This study protocol was reviewed and approved by ethics committees at each of the participating sites. This full list of participating sites and ethics committees can be found at the references [10, 11, 12].

## Consent

Written informed consent was obtained from participants prior to the study.

## Conflicts of Interest

The authors declare no conflicts of interest.

## Data Availability

The data that support the findings of this study are not publicly available due to privacy reasons but are available from the corresponding author upon reasonable request.
